# Functional Analysis of the 5′ Genomic Sequence of a Bovine Norovirus

**DOI:** 10.1371/journal.pone.0002169

**Published:** 2008-05-14

**Authors:** Omar Salim, Ian N. Clarke, Paul R. Lambden

**Affiliations:** Molecular Microbiology Group, University of Southampton Medical School, Southampton General Hospital, Southampton, United Kingdom; Yale University, United States of America

## Abstract

**Background:**

Jena Virus (JV), a bovine Norovirus, causes enteric disease in cattle and represents a potential model for the study of enteric norovirus infection and pathogenesis. The positive sense RNA genome of JV is organised into ORF1 (non-structural proteins), ORF2 (major capsid protein) and ORF3 (minor capsid protein). The lack of a cell culture system for studying JV replication has meant that work to date has relied upon *in vitro* systems to study non-structural protein synthesis and processing.

**Principal Findings:**

Only two of the three major ORF1 proteins were identified (p110 and 2C) following *in vitro* translation of JV RNA, the N-term protein was not detected. The N-term encoding genomic sequence (5′GS) was tested for IRES-like function in a bi-cistronic system and displayed no evidence of IRES-like activity. The site of translation initiation in JV was determined to be at the predicted nucleotide 22. Following the insertion of an epitope within the 5′GS the JV N-term protein was identified *in vitro* and within RNA transfected cells.

**Conclusions:**

The *in vitro* transcription/translation system is currently the best system for analysing protein synthesis and processing in JV. Unlike similarly studied human noroviruses JV initially did not appear to express the N-terminal protein, presenting the possibility that the encoding RNA sequence had a regulatory function, most likely involved in translation initiation in an IRES-like manner. This was not the case and, following determination of the site of translation initiation the N-term protein was detected using an epitope tag, both *in vitro* and *in vivo*. Although slightly larger than predicted the N-term protein was detected in a processed form *in vivo*, thus not only demonstrating initial translation of the ORF1 polyprotein but also activity of the viral protease. These findings indicate that the block to noroviral replication in cultured cells lies elsewhere.

## Introduction

Jena virus, a bovine norovirus, is a member of the *Caliciviridae* family of positive sense RNA viruses and was first isolated from the diarrhoeic stools of newborn calves [1], [2]. JV is a type I genogroup III (GIII) norovirus which is closely related to the type II GIII bovine noroviruses Newbury agent 2 and Dumfries [3], [4]. The GIII noroviruses are responsible for causing enteric disease in cattle [2], [5] and, thus, likely share a similar tissue tropism to the human-associated enteric noroviruses. Like human noroviruses [6] bovine noroviruses have a high seroprevalence [4]. JV is therefore a potentially useful model for studying the molecular biology of enteric norovirus pathogenesis and replication.

The 7.3 kb polyadenylated RNA genome of JV has been characterised previously [7] and, like other noroviruses, is organised into 3 open reading frames (ORFs). ORF1 encodes the non-structural proteins in the form of a large 185 kDa polyprotein, which is subsequently cleaved into functional replication proteins by the viral encoded 3C-like protease. ORF2 encodes the structural capsid protein (56 kDa) and ORF3 encodes a small basic protein, which has been shown to function as a minor capsid component [8]. JV ORF1 is consistent with other caliciviruses in that it encodes a 39 kDa 2C-like nucleoside triphosphatase (NTPase), a 3C-like protease and a 56 kDa 3D-like RNA-dependent RNA polymerase [7], [9]–[13]. However, the genomic sequence within the 5′ region of JV ORF1 (5′GS) displays a high level of divergence. This divergence is mainly attributed to the presence of several proline-encoding polypyrimidine tracts within the region predicted to encode a 35 kDa N-terminal protein [7]. The predicted size of N-terminal proteins relative to the size of the respective 2C proteins differs within the norovirus genus. Within the GI noroviruses, such as Southampton virus, the N-terminal protein (44.8 kDa) is larger in size compared to the 2C protein (39.6 kDa). This is in contrast to the GII noroviruses, such as Lordsdale virus and Camberwell virus, in that the N-terminal protein is smaller in size compared to the 2C protein [11], [14]. This is also the case for Jena virus in which the predicted JV N-terminal protein (35 kDa) is smaller than the JV 2C protein (39 kDa) [7].

The norovirus N-terminal protein varies in relative size across the genus, and the encoding sequence bears no similarity to other cellular or viral proteins. Alignment of the N-term protein sequences of various noroviruses indicates little similarity between genogroups within the first 180 residues, however towards the C-terminal end of the protein similarity between the amino acid residues increases. Recent studies investigating the functions of the Norwalk virus N-terminal protein have successfully demonstrated association with the Golgi apparatus in transfected cells [15]. In addition this study also identified a picornaviral 2B like region within the N-terminal protein, suggesting that the protein is involved with host cell membrane interactions, reinforcing other findings that have suggested that the Norwalk virus N-terminal protein disrupts intracellular protein trafficking, including proteins destined for the host cell membrane [16]. A 3C protease-mediated cleavage event within the N-terminal protein (37 kDa) was described for Camberwell virus, a genogroup 2 norovirus, yielding proteins of 22 kDa and 15 kDa [17]. Based on these observations and location within the genome it was hypothesised that the N-terminal protein of noroviruses corresponds to the 2AB region in picornaviruses.

Another possibility is that the N-term encoding RNA itself serves to function as a translational enhancer by interacting with cellular proteins involved in translation. Indeed, this phenomenon has been previously reported for Norwalk virus, within which a double stem loop structure has been predicted at the 5′ end of the genomic RNA [18]. It was subsequently demonstrated that elements within the 5′ end of Norwalk virus bind specifically with cellular proteins such as La, PTB and PCBP2 [19] which have all been implicated in IRES-mediated cap-independent translation in the closely related picornaviruses [20]–[23]. In this study the role of the JV 5′GS was investigated, including its potential to direct cap-independent translation initiation. The precise location of translation initiation in JV was also investigated.

## Results and Discussion

### JV ORF1 Polyprotein Processing

Previous studies of norovirus polyprotein processing have yielded three major products following *in vitro* transcription and translation, representing the uncleaved 3ABCD, N-term and 2C proteins. However, initial analysis of JV polyprotein processing indicated that only two major proteins are synthesised initially which, based on molecular weight predictions, are the 3ABCD (110 kDa) and the 2C (39 kDa). The lack of an N-terminal protein encoded by the JV 5′GS, predicted to be 35.3 kDa, is unique among the noroviruses that have been studied in this way. The *in vitro* transcription and translation profile for JV was therefore studied in more detail. As initial experiments had analysed TNT® reactions following a 1 hr incubation, reaction aliquots were harvested at time points before and after the recommended 1 hr incubation.

**Figure 1:**
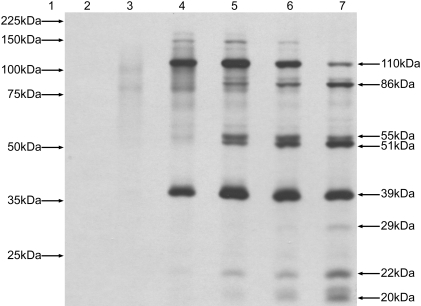
TNT® time course for JV displaying the progressive stages of post translational polyprotein processing. Molecular weight marker is represented by lane 1. Lanes 2–7 represent the following time points; 15 min, 30 min, 1 hr, 2 hr, 4 hr and 24 hr respectively.

The results in Figure 1 show that there are no major reaction products synthesised prior to the 1 hr time point, at which time the 3ABCD/p110 and 2C/p39 proteins are clearly visible. Extended incubation past the 1 hr point resulted in further proteolytic cleavage of p110 that coincided with the appearance of proteins of the following sizes: 86 kDa, 55 kDa, and 51 kDa. In addition proteins of 29 kDa, 22 kDa and 20 kDa were also visible at the 24 hr time point (Figure 1, lane 7). The only protein that was consistently visible following the 1 hr time point was the 2C/p39 protein. Despite prolonged incubation there was no indication that the N-terminal/p35 protein was synthesised.

A comprehensive study of polyprotein processing within the murine norovirus (MNV) suggests likely identities for the equivalent proteins in the similar profile for JV [12]. Using region specific antisera the authors were able to identify p110 as the 3ABCD uncleaved precursor, p90 as the 3BCD, p57.5 as the 3D-like polymerase, p52 as a 3ABC precursor and p40 as the 2C-like NTPase, which was determined by mutagenesis and microsequencing experiments. The 19 kDa protein was identified as the 3C-like protease. The antisera used to detect the MNV N-term protein recognised 3 products; one was the predicted molecular weight at 39 kDa and the other two bands migrated as a 45 kDa doublet.

### Assessment of translational enhancing potential of the JV 5′GS

The 5′GS region of JV is highly divergent compared to other noroviruses, mainly due to the relatively high cytosine content (32%), which contributes to an overall G/C content of 58%. There are many polypyrimidine tracts within the sequence, potentially yielding a relatively high degree of RNA secondary structure. Previous studies have described potential secondary RNA structure and interaction with proteins involved with IRES-mediated translation within the 5′ genomic region of Norwalk virus [18], [24]. It was of interest therefore, based on these findings, to ascertain whether or not the 5′GS of JV possessed IRES-like properties within the context of a ‘Bi-cistronic’ expression system, independently of other viral proteins, including the VPg which, in other caliciviruses, has been shown to be associated with translation initiation factors [25], [26].

Traditionally the bi-cistronic vector system has been used to define potential IRES-like sequences from a variety of viral and cellular mRNAs, and is recognized as being the standard test for this function [27]. A bi-cistronic vector is comprised of a 5′ and 3′ cistron; translation of the 5′ cistron being cap-dependent and translation of the 3′ cistron regulated by the putative IRES-like sequence. Thus, if the 3′ cistron is translated in addition to the 5′ cistron then the sequence of interest is said to have IRES-like properties, as translation is initiating internally.

To test for IRES-like function in JV, bi-cistronic constructs were made with a cap dependent 5′ EGFP cistron and a 3′ *lac*Z cistron under the translational control of either the JV 5′GS (pEGFP-C1/JV5′GS/lacZ) or an authentic EMCV IRES (pEGFP-C1/IRES/*lac*Z). CRFK cells were transfected with the bi-cistronic constructs and, following incubation, were assayed for EGFP and *lac*Z expression.

**Figure 2:**
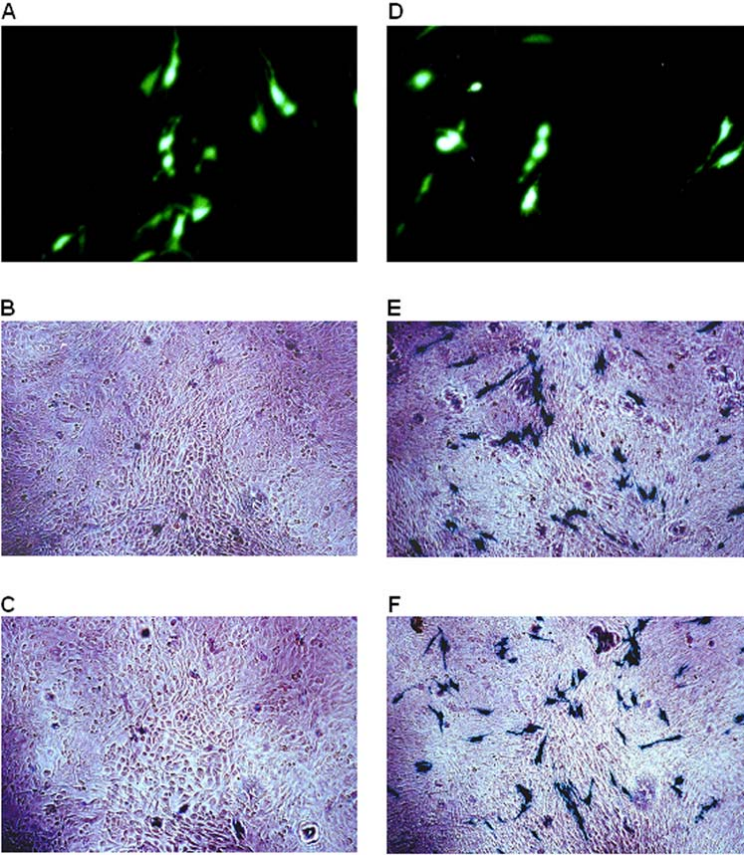
Fluorescence microscopy images and light microscopy images following x-gal staining of CRFK cells transfected with: A and B–pEGFP-C1/JV 5′ GS/*lac*Z, C–no DNA, D and E–pEGFP-C1/IRES/*lac*Z, F–pSV β-Gal reporter.

Both constructs were able to direct translation of the EGFP cistron effectively as expected (Figure 2a and Figure2d). The use of an authentic EMCV IRES to direct translation of the *lac*Z cistron was also effective (Figure 2e), with levels of β-galactosidase activity comparable to those of the β-galactosidase reporter (Figure 2f). However, no β-galactosidase activity was detected from cells transfected with the pEGFP-C1/JV5′GS/lacZ construct (Figure 2b), demonstrating that the JV 5′GS was unable to initiate translation, and therefore, in this context, did not possess any IRES-like functions.

### Translation initiation in JV ORF1

**Figure 3:**
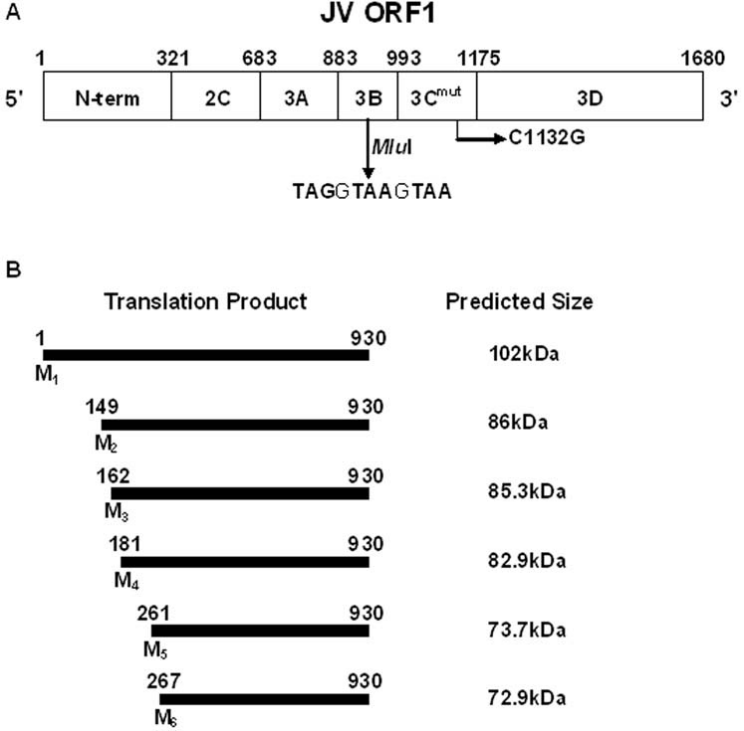
A-JV ORF1 polyprotein showing mutation to the 3C protease at amino acid 1132 and insertion of the polySTOP cassette at amino acid 930, as found in the JV 3C^mut^/polySTOP construct. B-The possible *in vitro* transcription and translation products from the 6 in-frame Met residues within the 5′GS and their respective predicted molecular weights. M_1_ represents the initiation codon found at nucleotide 22 within the JV genome.

**Figure 4:**
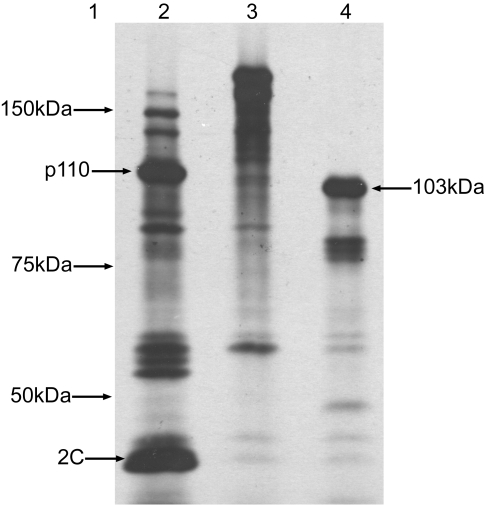
Analysis of TNT® products for JV (lane 2), JV 3C^mut^ (lane 3) and JV 3Cmut/polySTOP (lane 4), on a 7% SDS PAGE gel. Molecular weight marker representation is displayed in lane 1. The major band of approximately 103 kDa in lane 4 is indicative of translation initiation at nucleotide 22.

As it was clear that the JV 5′GS did not posses any IRES-like functions it was necessary to determine the location of translation initiation within ORF1. This was predicted be the ATG encoding methionine at nucleotide position 22, as it is situated in a favourable context for translation initiation [7]. To investigate this multiple translation termination codons (polySTOP) were inserted into the JV genome within the 3B-encoding region, downstream of the 5′GS, to halt translation at a defined point. *In vitro* transcription and translation of this construct would, in theory, yield a product whose size would relate to the initiation codon used within the 5′GS (Figure 3). To address the unlikely event of translation read-through or re-initiation downstream of the polySTOP, which would result in subsequent translation of the 3C protease and cleavage of the truncated ORF1 polyprotein, a mutation was made within the active site encoding region of the 3C protease within JV ORF1, to prevent any viral mediated cleavage of ORF1 translation products (JV 3C^mut^/polySTOP). A point mutation of the critical cysteine residue within the highly conserved GDCG motif to a glycine residue was performed, and this approach has been described for the successful inactivation of other norovirus' 3C activity [28]. *In vitro* transcription and translation analysis was performed on JV wild type (Figure 4, lane 2), JV 3C^mut^ (Figure 4, lane 3) and JV 3C^mut^/polySTOP (Figure 4, lane 4). The mutation of the critical cysteine residue within the 3C region of JV successfully inactivated the 3C protease, thus a large, >200kDa uncleaved polyprotein is yielded following TNT®. The major product generated by JV 3C^mut^/polySTOP was calculated to be 103kDa in size. Based on computer predictions this is in agreement with the initiation of translation occurring at nucleotide 22, which demonstrates that the JV N-term protein is translated in full *in vitro*. At this time it is not possible to determine whether translation of intracellular VPg-bound viral RNA initiates at nucleotide 22, although it is likely given the favourable context in which the initiation codon is situated.

### Immune detection of JV N-term

As the JV N-term was found to be translated *in vitro* attempts were made to express and purify the protein in bacteria for immunisation so that the protein could be identified by radio-immune precipitation assay (RIPA), as it was possible that the N-term protein was migrating on gels aberrantly and possibly co-migrating with 2C. Attempts to express the protein in bacteria were unsuccessful due to toxicity. Therefore, the 14aa V5 epitope encoding sequence was cloned in frame into the JV cDNA construct at nucleotide position 123 (JV V5). The V5 epitope originates from the P and V proteins of the SV5 paramyxovirus [29], for which a commercially available monoclonal antibody is used for detection.

**Figure 5:**
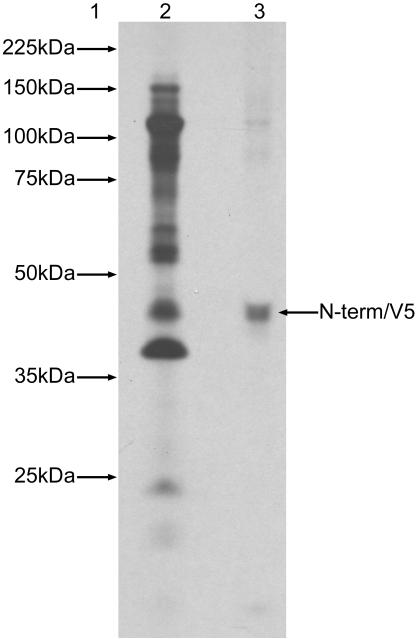
Analysis of TNT® (lane 2) and RIPA (lane 3) products for JV/V5. The N-term/V5 product is approximately 42 kDa in size, larger than the predicted size of 35.3 kDa. Molecular weight marker is represented in lane 1.

Following *in vitro* transcription and translation of JV V5 a new product, approximately 42 kDa in size, was visible (Figure 5, lane 2). This product was not observed in any prior analyses of JV. To confirm that this protein was V5/N-term associated the TNT® reaction was subjected to RIPA using the anti-V5 antibody (Figure 5, lane 3). This confirmed expression of the N-term protein *in vitro*.

**Figure 6:**
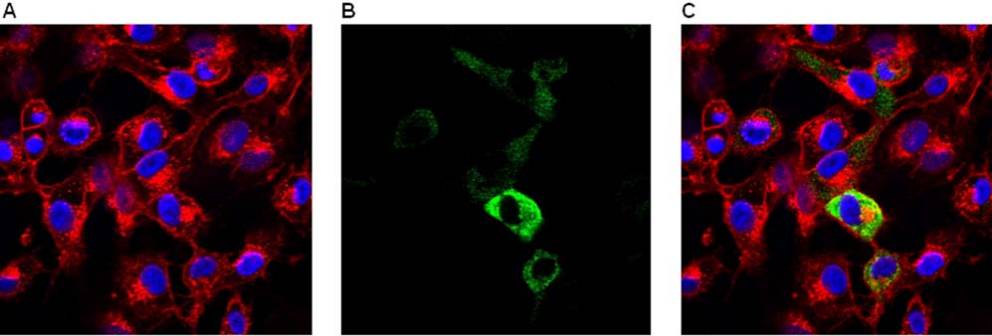
Confocal immunofluorescence of CRFK cells transfected with JV/V5 RNA. A = cells stained with wheat germ agglutinin (plasma and Golgi/ER membrane marker, red) and DAPI (blue). B = cells stained with anti-V5 (green). C = merged.

To confirm expression of the V5/N-term protein in cell culture capped RNA was synthesised from the JV V5 T7 cDNA construct, which was used to transfect CRFK cells. As there is currently no host cell line in which to propagate JV the CRFK cell line was used as it has been shown to support the replication of feline calicivirus [30]. Confocal immunofluorescence of transfected cells using the anti-V5 antibody demonstrated expression of the V5/N-term protein in cultured cells (Figure 6). Expression of the V5/N-term protein was diffuse and did not co-localise with the Golgi/ER/plasma membrane marker wheat germ agglutinin (WGA) and therefore displays a different pattern of cellular expression compared to Norwalk virus [15]. Cells transfected with the wild type full length JV RNA were negative for fluorescence (data not shown).

**Figure 7:**
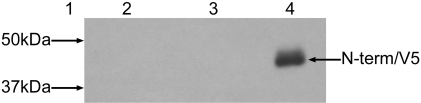
Western analysis from lysates of CRFK cells transfected with no RNA (lane 2), JV RNA (lane 3) and JV/V5 RNA (lane 4) using the anti-V5 monoclonal antibody. Bradford analysis was performed on the lysates to ensure equal loading. The appearance of the 42 kDa V5 product confirms ORF1 translation and activity of the 3C protease *in vivo*. Molecular weight marker is represented in lane 1.

Lysates of cells transfected with wild type JV and JV/V5 RNA were subjected to western blot using the anti-V5 antibody (Figure 7). No product was present for cells transfected with wild type JV RNA, but a protein of approximately 42 kDa in size was visible in cells that had been transfected with JV/V5 RNA, confirming N-term expression and size as seen in the *in vitro* system. In addition, this important observation also confirms for the first time that the JV 3C protease was active in cells transfected with capped RNA as the size of the V5/N-term indicated successful cleavage of the protein from the ORF1 polyprotein.

**Figure 8:**
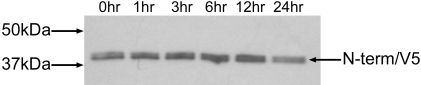
Degradation analysis of N-term/V5 following treatment of JV/V5 transfected CRFK with cycloheximide (CHX). Whole cell lysate was collected at the following time points following CHX treatment: 0 hr, 1 hr, 3 hr, 6 hr, 12 hr, 24 hr. Bradford analysis was performed on the lysates to ensure equal loading. Following Western analysis the ECL treated membrane was exposed to film for 1 min. Molecular weight marker is represented in lane 1.

To address the issue of potential rapid degradation of the JV N-term protein CRFK cells were transfected with JV V5 RNA and were harvested at designated time points following the addition of the protein synthesis inhibitor cycloheximide. Cell lysates were analysed by Western blot using the anti-V5 antibody (Figure 8). The consistent appearance of the N-term/V5 protein suggested that it is stable and insensitive to degradation by viral and host cell proteases.

The predicted molecular weight of the JV N-term is 35.3 kDa, based on the site of initiation of translation and location of conserved cleavage sites. The appearance, therefore, of a previously unseen 42 kDa protein in the *in vitro* transcription and translation profile was unexpected but this protein does represent a translation product for the JV 5′GS. To date, it has not been possible to explain the difference in the predicted and observed sizes for the JV N-term, and the addition of the 14 amino acid V5 epitope within JV N-term does not account for this apparent large shift in molecular weight. However, a recent study described a similar anomaly when investigating proteolytic processing in the murine norovirus MNV-1 [12]. The predicted molecular weight for the MNV-1 N-term protein was 38.3 kDa. The authors successfully generated antisera against the MNV-1 N-term and used it to immunoprecipitate the protein from *in vitro* transcription and translation reactions and observed that the N-term existed as a 45 kDa doublet, in addition to the predicted size of 38 kDa. However, when MNV-1 N-term antisera was used to probe MNV-1-infected cell lysates only the 43–45 kDa doublet and a large 115 kDa precursor could be detected, suggesting that the predicted 38 kDa form of the N-term is not generated in cell culture. Again, it was not possible to conclusively determine the cause of this discrepancy, but it was speculated that the N-term protein may migrate abnormally in SDS-PAGE, or may be proteolytically processed at a previously unknown cleavage site downstream of the protein's predicted C-terminus. It is also possible that the N-term protein might be modified in some way leading to a shift in observed molecular weight. At this time the same conclusions would seem appropriate for the JV N-term. In addition, it is not known why the JV N-term was previously not detected in *in vitro* transcription and translation studies prior to the insertion of the V5 epitope. It cannot be ruled out, however, that the wild type JV N-term aberrantly co-migrates with the 39 kDa JV 2C protein in SDS-PAGE. Indeed, the appearance of the V5/N-term product from transfected cell lysates would appear to be one of a doublet (Figure 8), also analogous to the observed appearance of the MNV N-term protein in infected cells, suggesting the likelihood of a further cleavage site within the JV N-term protein which has yet to be elucidated. Nevertheless, these studies clearly demonstrate that a protein representative of the 5′GS of JV is translated both *in vitro* and *in vivo* and is proteolytically processed from the ORF1 polyprotein following translation initiation at nucleotide 22.

### Conclusions

Human norovirus infection has been shown to be the leading cause of non-bacterial gastroenteritis [31], however there is currently no cell culture system available to facilitate viral replication and ethical considerations have hindered progress in establishing a permissive human organ culture system. The study of Jena virus offers a potential animal model of enteric noroviral infection. However, until a permissive bovine cell and/or organ culture systems is established analysis of the molecular mechanisms underpinning viral replication and pathogenesis rely upon *in vitro* systems, most notably polyprotein synthesis and processing.

Unlike similarly studied human noroviruses JV initially did not appear to express the N-terminal protein, presenting the possibility that the encoding RNA sequence had a regulatory function itself, most likely involved in translation initiation in an IRES-like manner. This was shown not to be the case and, following determination of the site of translation initiation at the predicted nucleotide 22 the N-term protein was detected following the insertion of an epitope tag, both *in vitro* and *in vivo*. Although slightly larger than predicted the N-term protein was detected in a processed form *in vivo*, thus not only demonstrating initial translation of the ORF1 polyprotein but also activity of the viral encoded protease. These important findings indicate that the block to replication of enteric norovirus in cultured cells cannot be attributed to a failure to synthesise and process the non-structural proteins. The detection of processed and active ORF1 proteins in transfected cultured cells, however, highlights the potential for the development of cell and bovine organ based systems to facilitate the replication of Jena virus.

## Materials and Methods

### Construction of Bi-cistronic vectors

The pEGFP-C1 vector (Clontech) comprises of an EGFP coding sequence under the control of a CMV promoter and a Kozak translation initiation site. Downstream of the EGFP sequence is the multiple cloning site containing unique *Bgl*II, *Sac*I, *Hin*dIII and *Apa*I restriction sites. Contruction of pEGFP-C1/JV 5′ GS/*lac*Z was as follows; The JV 5′ GS sequence was amplified from the JV full length cDNA clone [7] using Bio-X-Act DNA polymerase (Bioline) with the primers 5′ GS F (5′-AACTGC**AGATCT** TAATAAGTGAATGAAGACTTTGACGAT-3′), containing the *Bgl*II restriction site (bold) and two in-frame translation termination codons (underlined) to ensure that translation of the EGFP sequence did not carry over to the 5′ GS, and 5′ GS R (5′-AACTGC**AAGCTT**CTGCAGGACACAATGAGG-3′), containing the*Hin*dIII restriction site. The JV 5′ GS amplicon was ligated to the pEGFP-C1 vector, following restriction enzyme digestion of both amplicon and vector with *Bgl*II and *Hin*dIII restriction enzymes, and the ligated DNA used to transform *E.coli* Top10 (Invitrogen). This intermediate construct was named pEGFP-C1/JV 5′ GS. The *lac*Z coding sequence was amplified from the pSV-β-Gal reporter vector (Promega) using Bio-X-Act DNA polymerase and the primers *lac*Z F (5′-AACTGC**AAGCTT** GATATGGGGGATCCCGTCGTTTTACAACG-3′), containing the *Hin*dIII restriction site (bold) and a kozak translation initiation site (underlined), and *lac*Z R (5′-AACTGC**GGGCCC** TTATTATTTTTGACACCAGACCA-3′) containing the *Apa*I restriction site (bold) and translation termination codons (underlined). The *lac*Z amplicon was ligated to the pEGFP-C1/JV 5′ GS vector following restriction enzyme digest of both amplicon and vector with *Hin*dIII and *Apa*I restriction enzymes, and the ligated DNA used to transform *E.coli* Top10. The construct was verified by sequencing. Construction of pEGFP-C1/IRES/*lac*Z was as follows; The EMCV IRES sequence was amplified from the pIRES2-EGFP vector (Clontech) using Bio-X-Act DNA polymerase and the primers IRES Bgl F (5′-ACTCGA**AGATCT** TAATAGAGCTTCGAATTCTGCAGTCGA-3′), containing the *Bgl*II restriction site (bold) and translation termination codons (underlined) to prevent carry over translation as before, and IRES Sac R (5′- ACTCGA**GAGCTC**TGTGGCCATATTATCATCGTG-3′), containing the *Sac*I restriction site (bold). The IRES amplicon was ligated to the pEGFP-C1 vector follwing restriction enzyme digest of both amplicon and vector with *BgI*II and *Sac*I restriction enzymes, and the ligated DNA used to transform *E.coli* Top10. The *lac*Z amplicon described previously was ligated to the intermediate pEGFP-C1/IRES vector following restriction enzyme digest of both amplicon and vector with *Hin*dIII and *Apa*I restriction enzymes, and the ligated DNA used to transform *E.coli* Top10.

### Construction of JV 3C^mut^ and JV 3C^mut^/polySTOP

The JV 3C protease mutant was created by point mutation of the critical TGT encoded cysteine residue, within the GDCG active site motif, to a GGT encoded glycine residue by mutagenic overlap PCR using Bio-X-Act DNA polymerase. Three rounds of amplification using the JV full length cDNA clone as template were used to generate the final mutant protease cassette. Round 1 used the primers JV F1 (5′-CGTCTCAGGGTTGATACT-3′) and JV Mut 1 (5′-GCAACCAC**C**GTCACCAG-3′), yielding a 222 bp amplicon (point mutation nucleotide shown in bold). Round 2 used the primers JV Mut 2 (5′-CTGGTGAC**G**GTGGTTGC-3′) and JV R2 (5′-TTCCTGGGAGGAACAAGTT-3′), yielding a 651 bp amplicon. Amplicons generated in rounds 1 and 2 were pooled to serve as template for round 3 using the primers JV NF (5′-ATGTCAACCACCACCAGC –3′) and JV NR (5′-AAGGGCTCCGGTGAAGG-3′). This cassette contained two *Bcl*I restriction sites flanking the 3Cprotease active site, as also found in the wild-type full length clone. Restriction digest using *Bcl*I was used to remove the appropriate wild-type cassette from the JV full length clone. The mutant cassette was also digested with *Bcl*I prior to ligation to the *Bcl*I-digested JV full length clone. The ligated DNA was used to transform *E.coli* Top10, and was designated JV 3C^mut^.

Construction of JV 3C^mut^/polySTOP was as follows: complementary oligonucleotides with three translation termination codons (underlined) in each reading frame in sense and anti-sense orientations were desgined in such a way that upon annealing the duplex would contain blunt termini. The oligonucleotides were termed pSTOP Top (5′-CTAGGTAAGTAA **ACGCGT**CTACTCACTCAC-3′) and pSTOP Comp (5′- GTGAGTGAGTAG **ACGCGT**TTACTTCAATAG-3′). Each oligo (1 µg) was incubated with T4 polynucleotide kinase and ATP to phosphorylate the 5′ termini, pooled and heated to 75°C for 15 min, and left to cool to room temperature to anneal the oligos. Following purification the polySTOP duplex was ligated to *Eco*47III digested JV 3C^mut^, and ligated DNA was used to transform *E.*coli Top10. The duplex contained the unique restriction site *Mlu*I (shown in bold) to assist screening of recombinant clones.

### Construction of JV V5

The V5 epitope (N-Gly-Lys-Pro-Ile-Pro-Asn-Pro-Leu-Leu-Gly-Leu-Asp-Ser-Thr-C) is recognized by the anti-V5 monoclonal antibody (Invitrogen). Complementary oligonucleotides encoding the V5 epitope were designed in such a way as to generate *Sac*II compatible termini following annealing (bold), and to preserve the reading frame when inserted into the *Sac*II restriction site at nucleotide 123 within the 5′ GS of the JV genome (underlined). The oligos were termed V5 Top (5′-GGTAAGCCTATCCCTAACCCTCTCCTCGGTCTCGATTCTACGA **GC**-3′) and V5 Comp (5′-TCGTAGAATCGAGACCGAGGAGAGGGTTAGGGATAGGCTTACC**GC**-3′). The oligos were phosphorylated and annealed as described previously and the duplex ligated to the *Sac*II digested JV full length clone. Ligated DNA was used to transform *E.coli* Top10.

### In vitro Transcription/Translation and RIPA analysis

*In vitro* coupled transcription and translation was performed using the TNT® Coupled Reticulocyte Lysate System (Promega) as per the manufacturer's instructions. Reactions were incubated at 30°C for 1–2 hr. For non-radiolabelled reactions the ^35^ S-Methionine was replaced with 1 mM unlabelled Methionine (2 µl). Reaction products (1–2 µl) were analysed by SDS-PAGE. Gels were stained and prepared for autoradiography by incubating for 30 min in a solution containing 32 g sodium salicylate, 100 ml methanol and 100 ml dH_2_O. Gels were dried under vacuum and the reaction products were detected by exposure to Kodak X-Omat scientific imaging film (Sigma) at −70°C for 16 hr followed by developing using a Kodak automated developer.

Specific V5-tagged proteins synthesised by TNT® were precipitated from 5–10 µl of reaction product using the anti-V5 monoclonal antibody (Invitrogen) at the recommended dilution in 600 µl of 1× RIPA buffer (diluted from 10x stock: 10 mM Tris-HCl (pH 7.5), 1 mM EDTA, 0.15 mM NaCl, 0.1% SDS, 0.5% Empigen BB, 0.1 mM phenylmethylsulphonylfluoride) for 1 hr at 37°C. This was followed by a second incubation of tube for 2 hr rotating at room temperature with goat anti-mouse immunoglobulin G agarose beads (Sigma) to absorb the immune complexes. The beads were washed three times with 500 µl 1× RIPA buffer and once with 500 µl PBS. The beads were resuspended in sample buffer for analysis by SDS-PAGE and autoradiography as before.

### Transfection of CRFK cells with Bi-cistronic vectors

Endotoxin-free preparations of plasmid DNA were prepared using the GenElute™ Endotoxin free plasmid midi prep kit (Sigma). Crandall-Reese Feline Kidney cells (CRFKs) were seeded into a 12 well tray at approximately 40–50% confluence. CRFK cells were transfected with no DNA (negative control), pSV-β-Gal (control for β-galactosidase activity), pEGFP-C1/JV 5′ GS/*lac*Z and pEGFP-C1/IRES/*lac*Z (control for IRES activity) using the Superfect™ transfection reagent (Qiagen) as per the manufacturer's recommendations. Following a 16 hour incubation the cells were observed for EGFP expression using a Leica Leitz DMRB fluorescence microscope. The cells were washed in PBS and fixed using a 0.5% solution of glutaraldehyde for 30 min at room temperature. The cells were incubated with an X-Gal stain solution: 5 mM K_3_Fe(CN)_6_, 5 mM K_4_Fe(CN)_6_, 2 mM MgCl_2_, 1x X-Gal (Sigma) for 4 hours at 37°C and were observed for β-glactosidase activity by light microscopy. The experiment was performed more than once to confirm the results.

### RNA synthesis and transfection of CRFK cells

JV V5 and JV FLC T7 cDNA plasmid constructs were linearised using *Nde*I (Invitrogen). Capped RNA was synthesised using the mMessage mMachine® Capped RNA Transcription kit (Ambion) according to the manufacturer's instructions. CRFK cells were seeded into 6 well trays at approximately 50% confluence and were transfected with 2 µg purified RNA per well using Transmessenger transfection reagent (Qiagen) according to the manufacturer's instructions.

### Immunological analysis

For immunofluorescence CRFK cells were seeded onto 19 mm coverslips in 6 well trays and were transfected with RNA as described. Following a 24 hr incubation the coverslips were washed with PBS and fixed in 4% formaldehyde for 15 min at room temperature. Cells were permeabilised and blocked in saponin buffer, also used as staining buffer, (0.1% saponin, 10% foetal calf serum, 0.1% sodium azide) for 1 hr at 4°C. Cells were stained using an anti-V5 monoclonal antibody (Invitrogen) followed by an anti-mouse Alexafluor 488 conjugated secondary antibody (Molecular Probes) at the recommended dilution in staining buffer for 30 min in the dark. Cells were then stained for 30 min in the dark with a Wheat Germ Agglutinin Alexafluor 594 nm conjugate (Molcular Probes) to allow identification of plasma and Golgi membranes. Coverslips were washed and mounted onto slides using Vectashield containing DAPI (Vector Labs). Microscopy was performed using an inverted Leica TCS-NT confocal laser scanning microscope.

The anti-V5 antibody was also used to detect V5-tagged protein by Western blot. Cell lysates were prepared following transfection using lysis buffer (0.15 M sodium chloride, 0.5% (v/v) sodium deoxycholate, 0.1% (w/v) SDS, 50 mM Tris-Cl pH 8.0) and protease inhibitor cocktail (Sigma). Lysates were incubated for 15 min on ice followed by sonication to shear genomic DNA. Following Bradford analysis equal protein content from JV V5 and JV FLC lysates were run on a 10% SDS-PAGE gel and subsequently transferred onto Immobilon-P PVDF membrane (Millipore) according to the manufacturer's recommendations. The membrane was probed using the anti-V5 monoclonal antibody at the manufacturer's recommended dilution, followed by an anti-mouse HRP-copnjugated secondary antibody (Santa Cruz) at the recommended diltution. The ECL Western blotting reagents kit (G.E. Healthcare) was used to detect antibody bound protein, which was visualised by exposure to BioMax Light film (Kodak).

### Degradation analysis

CRFK cells were seeded into 6 well trays and transfected with capped JV V5 RNA as described. Following a 24 hr incubation cycloheximide (Sigma) was added to the cells at a final concentration of 50 µg/ml. Cells were harvested at indicated times for the preparation of lysates for V5 Western analysis as described above. Bradford reagent (Sigma) was used to ensure equal loading of lysates according to the manufacturer's recommendations.
